# Angiopoietin-like 3 inhibition of endothelial lipase is not modulated by angiopoietin-like 8

**DOI:** 10.1016/j.jlr.2021.100112

**Published:** 2021-08-27

**Authors:** Kelli L. Sylvers-Davie, Ashley Segura-Roman, Alicia M. Salvi, Kylie J. Schache, Brandon S.J. Davies

**Affiliations:** Department of Biochemistry and Molecular Biology, Fraternal Order of Eagles Diabetes Research Center, and Obesity Research and Education Initiative, University of Iowa, Iowa City, IA, USA

**Keywords:** endothelial lipase, lipoprotein lipase, HDL, lipoprotein metabolism, endothelial cells, angiopoietin-like proteins, ANGPTL3, ANGPTL8, triglycerides, cardiovascular disease, ANGPTL, angiopoietin like, ANGPTL3, angiopoietin-like 3, ANGPTL8, angiopoietin-like 8, cDNA, complementary DNA, CHO, Chinese hamster ovary, EL, endothelial lipase, HEK 293T, human embryonic kidney 293T cells, HSPG, heparan sulfate proteoglycan, TG, triglyceride, RHMVEC, rat heart microvessel endothelial cell

## Abstract

High plasma triglyceride (TG) levels and low HDL-C levels are risk factors for atherosclerosis and cardiovascular disease. Both plasma TG and HDL-C levels are regulated in part by the circulating inhibitor, angiopoietin-like 3 (ANGPTL3). ANGPTL3 inhibits the phospholipase, endothelial lipase (EL), which hydrolyzes the phospholipids of HDL, thus decreasing plasma HDL levels. ANGPTL3 also inhibits LPL, the lipase primarily responsible for the clearance of TGs from the circulation. Previous studies have shown that ANGPTL3 requires complex formation with the related ANGPTL protein, angiopoietin-like 8 (ANGPTL8), to efficiently inhibit LPL, but the role of ANGPTL8 in EL inhibition is not known. In this study, we characterized inhibition and binding of EL by ANGPTL3 and investigated the role of ANGPTL8 in EL inhibition. We found that inhibition of EL by ANGPTL3 was dose dependent and temperature dependent. Interestingly, this inhibition was diminished when EL was bound to endothelial cells or in the presence of heparin. Unlike previous findings with LPL, we found that ANGPTL8 did not significantly alter the binding or the inhibition of EL by ANGPTL3. In addition, we found that a common ANGPTL8 variant, which encodes an R59W mutation, altered the ability of ANGPTL3 to bind and inhibit LPL but not EL. Together, our data indicate that ANGPTL8 is not necessary for EL inhibition. We conclude that ANGPTL8 is specific for the regulation of TG-rich lipoproteins through the LPL pathway and that therapeutically targeting ANGPTL8 for the treatment of hypertriglyceridemia or cardiovascular disease may have different outcomes than targeting ANGPTL3.

Misregulation of lipid hydrolysis is associated with a variety of metabolic diseases, including coronary heart disease and atherosclerosis. Low HDL-C levels often correlate to reduced cardiovascular health and a higher risk for cardiovascular events (1, 2, 3, 4). Plasma HDL-C levels are regulated in part by the enzyme, endothelial lipase (EL) (5, 6). In mice, EL overexpression decreases plasma HDL-C and HDL phospholipid levels, whereas increased HDL-C and HDL phospholipid levels are observed in EL knockout mice (5, 7, 8). EL is secreted into the vasculature by endothelial cells in vascularized tissues, such as the lung, liver, and kidney (5, 9). Once secreted, EL hydrolyzes the phospholipids of HDL, leading to lower circulating HDL-C levels (5, 7). EL appears to also hydrolyze the phospholipids of VLDL remnant particles and may thus modulate the conversion of VLDL remnants to LDL (10).

Three members of the angiopoietin-like (ANGPTL) family of proteins have been shown to regulate lipid metabolism. Angiopoietin-like 3 (ANGPTL3), angiopoietin-like 4 (ANGPTL4), and angiopoietin-like 8 (ANGPTL8) have all been shown to regulate plasma triglyceride (TG) levels through their inhibition of LPL (11, 12, 13, 14, 15, 16, 17). ANGPTL3 has also been shown to regulate EL (18). In vitro, ANGPTL3 inhibits EL activity in a dose-dependent manner (18). Moreover, plasma HDL-C levels are decreased in ANGPTL3-deficient mice (18). Re-expression of ANGPTL3 in ANGPTL3-knockout mice increases plasma HDL-C levels (18). Together, these studies indicate that ANGPTL3 modulates HDL-C levels by targeting EL. However, the mechanism by which ANGPTL3 inhibits EL has not been characterized.

EL belongs to the same lipase family as LPL, and the two share 44% protein identity. Despite this identity, EL and LPL have different substrate specificities, with EL targeting the phospholipids of HDL (5, 6) and LPL targeting TGs within TG-rich lipoproteins (19, 20). Efficient inhibition of LPL by ANGPTL3 requires that ANGPTL3 forms a complex with ANGPTL8 (15, 21, 22, 23). It is not yet known if ANGPTL8 is also required for, or modulates, the inhibition of EL by ANGPTL3.

In this study, we further characterize the ability of ANGPTL3 to inhibit EL in solution and when bound to the surface of endothelial cells. We also evaluate the effect of ANGPTL3 cleavage on this inhibition. Finally, we test the role of ANGPTL8 in the inhibition of EL by ANGPTL3 and test a common ANGPTL8 variant associated with lower HDL-C levels for changes in its ability to regulate both EL and LPL.

## Materials and methods

### Expression constructs

The generation of plasmid constructs expressing strep-tagged mouse ANGPTL3 (pHS18), strep-tagged and smallBiT-tagged mouse ANGPTL3 (pEB14), a truncated (amino acids 1–221) strep-tagged mouse ANGPTL3 (pEB2), and V5 and 6×His-tagged mouse ANGPTL8 (pWL1) have been described previously (21).

A truncated (amino acids 1–121) ANGPTL3 with a C-terminal strep tag and a C-terminal smallBiT tag was generated by deleting the coding sequence for amino acids 222–455 from pEB14 using site-directed mutagenesis. Plasmid constructs expressing cleavage-resistant strep-tagged mouse ANGPTL3 (pHS21) and cleavage-resistant strep-tagged and smallBiT-tagged mouse ANGPTL3 (pKS12) were generated from pHS18 and pEB14, respectively, by mutating the conserved cleavage motif ^221^RAPR^224^ to ^221^GSGS^224^ using site-directed mutagenesis. Expression constructs for strep-tagged mouse ANGPTL3 and strep-tagged and smallBiT-tagged mouse ANGPTL3 containing N48A, Q53A, and H55A mutations (pBD220 and pAS6, respectively) were generated from pHS18 and pEB14 using site-directed mutagenesis. A V5-tagged and 6×His-tagged mouse ANGPTL8 containing an R59W mutation (pKS29) was generated by introducing a C175T and a C177G substitution into the ANGPTL8 coding region of pWL1 using site-directed mutagenesis. To generate a smallBiT-tagged control for NanoBiT experiments (pAS7), we used site-directed mutagenesis to add a strep and smallBiT tag to a construct expressing human alpha-1-antitrypsin.

A lentiviral construct expressing untagged human EL (pKS7) was generated by amplifying the coding sequence of human EL from complementary DNA (cDNA) (LIPG; MGC MHS6278-202806078) and inserting it into the lentiviral vector pRRL-cPPT-MCS-IRES using InFusion cloning (Clontech). Lentiviruses containing this construct were produced by transfecting human embryonic kidney 293T (HEK 293T) cells with pKS7 and the lentiviral packaging vectors pMD2.G (Addgene; #12259), pRSV-Rev (Addgene; #12253), and pMDLg/pRRE (Addgene; #12251). Conditioned media containing lentiviruses with the EL construct were collected and concentrated using Lenti-X Concentrator (Clontech; catalog no. 631231). pKS7-lentiviruses were used to generate cell lines as described later. A plasmid construct expressing LargeBiT EL (pLB1) was generated by first amplifying the coding sequence of human EL from cDNA and inserting it into a pCDNA6 vector using InFusion cloning. The LargeBiT tag was then amplified from a pre-existing construct (pEB12 (21)) and inserted at the C-terminus of EL using InFusion cloning. A lentiviral construct expressing FLAG-tagged human LPL (pAH1) was generated by cloning the coding sequence of LPL-FLAG from pSS1 (24) to the lentiviral vector pRRL-cPPT-MCS-IRES.

### Cell lines

Rat heart microvessel endothelial cells (RHMVECs; VEC Technologies, Inc) were grown in MCDB-131 base medium (GenDEPOT) supplemented with 10 mM l-glutamine, 1% penicillin/streptomycin antibiotic solution (10,000 U/ml penicillin and 10,000 μg/ml streptomycin; Gibco), 5% fetal bovine serum (Atlanta Biologicals), 1 μg/ml hydrocortisone (Sigma-Aldrich), 10 μg/ml human epidermal growth factor (Gibco, Life Technologies), and 12 μg/ml bovine brain extract (Lonza). To generate a stable cell line expressing human EL, 80% confluent RHMVECs in 6-well plates were transduced with human EL (pKS7) lentiviruses with 4 μg/ml Polybrene (#134220; Santa Cruz Biotechnology) in a total volume of 1 ml DMEM. Twenty-four hours post-transduction, cells were washed with PBS and incubated in MCDB-131 complete medium for 48 h. Cells were then subjected to selection with 6 μg/ml puromycin for 5 days. A stable cell line of RHMVECs expressing GPIHBP1 was generated by transducing cells with a lentiviral GPIHBP1 construct described previously (24).

HEK 293T cells were grown in DMEM supplemented with 5% FBS, 1% penicillin/streptomycin antibodies, and 1% l-glutamine (complete DMEM). To generate stable cell lines expressing human EL, 80% confluent HEK 293T cells were transduced with lentiviruses coding for human EL (pKS7) using 4 μg/ml Polybrene in a total volume of 1 ml DMEM in 6-well plates. Twenty-four hours post-transduction, cells were washed with PBS and incubated in complete DMEM for 48 h, then subjected to selection with 3 μg/ml puromycin for 5 days. Stable HEK 293T cell lines expressing V5-tagged ANGPTL8 or LargeBiT-FLAG-tagged LPL (pEB12) generated by lentivirus transduction were described previously (21).

Chinese hamster ovary (CHO) cells were grown in F12 medium supplemented with 5% FBS, 1% penicillin/streptomycin antibodies, and 1% l-glutamine (complete F12). To generate a stable cell line expressing FLAG-tagged LPL, 80% confluent CHO cells were transduced with lentiviruses coding for FLAG-tagged LPL (pAH1) using 4 μg/ml Polybrene in a total volume of 1 ml DMEM in 6-well plates. Twenty-four hours post-transduction, cells were washed with PBS and incubated in complete F12 medium for 48 h and then subjected to selection with 10 μg/ml puromycin for 5 days.

### Production and quantification of ANGPTL conditioned media

For all ANGPTL3 and smallBiT proteins, HEK 293T cells were grown to 80% confluency in T75 flasks in complete DMEM. Cells were transfected with 10 μg of DNA (ANGPTL3 or ANGPTL3 + ANGPTL8) and 20 μl of 1 mg/ml polyethylenimine. Cells were switched to serum-free DMEM and 1× ProteaseArrest protease inhibitor cocktail (APExBIO) 24 h post-transfection. Conditioned media were collected 48–72 h later. To produce 293T control media, HEK 293T cells were transfected and media were collected in the same manner; only no DNA was added to the transfection. This control conditioned media were used as a control for all experiments involving ANGPTL conditioned media. The concentration of ANGPTL3 in conditioned media was determined using a mouse ANGPTL3 ELISA kit (RayBiotech; catalog no. ELM-ANGPTL3-1) following the manufacturer's instructions and using purified recombinant ANGPTL3 to generate a standard curve. To produce conditioned media containing ANGPTL8 without ANGPTL3, HEK 293T cells stably expressing ANGPTL8 were cultured in serum-free DMEM for 72 h, and conditioned media were then collected. The concentration of mouse ANGPTL8 protein in the conditioned media was determined by quantitative Western blotting using known concentrations of purified recombinant ANGPTL8 protein produced in bacteria as a standard as we have done previously (21).

### Production of EL and LPL conditioned media

To produce EL conditioned media, HEK 293T stably expressing human EL were grown to 80% confluency in DMEM supplemented with 5% FBS, penicillin/streptomycin antibiotics, and l-glutamine. Media were changed to serum-free OptiMEM containing 1× ProteaseArrest and 0.1 U/ml heparin (Fresenius Kabi USA, LLC). EL conditioned media were collected after 24 h. Protein expression was confirmed by Western blotting using a mouse monoclonal against EL (LIPG antibody clone 3C7; Lifespan Biosciences; diluted 1:3,000), and activity was tested using a EnzChek™ Molecular Probes™ Phospholipase A1 Assay Kit (Invitrogen). Conditioned media from cells not expressing EL had little detectable phospholipase A1 activity (supplemental Fig. S1). To produce LargeBiT-tagged EL, HEK 293T cells were grown to 80% confluency in T75 flasks in complete DMEM. Cells were transfected with 10 μg of pLB1 and 20 μl of 1 mg/ml polyethylenimine. Cells were switched to serum-free OptiMEM containing 1× ProteaseArrest and 0.1 U/ml heparin 24 h post-transfection. Conditioned media were collected 24 h later. EL concentration was determined via Western blot using a standard curve of strep-tagged EL with a known concentration. Western blots were performed using a mouse anti-LIPG antibody at 1:3,000 dilution (Lifespan Biosciences).

To produce LPL conditioned media, CHO cells stably expressing FLAG-tagged LPL were grown to 90% confluency and then switched to serum-free F12 media supplemented with 1% penicillin/streptomycin antibiotics, and 1% l-glutamine containing 1× ProteaseArrest. After 72 h, the conditioned media were concentrated 10× using Amicon Ultra-15 centrifugal filter units (EMD Millipore; #UFC901024). LPL activity was assessed by a lipase activity assay (see later). LargeBiT LPL (pEB12) was produced as described previously (25). Briefly, HEK 293T cells stably expressing LargeBiT LPL were grown to confluence and then switched to serum-free DMEM containing 1× ProteaseArrest. After 48 h, the conditioned media were collected and concentrated 10× using Amicon Ultra-15 centrifugal filter units. The presence of LargeBiT LPL was detected by Western blot using a mouse anti-FLAG antibody at 1:5,000 dilution (Sigma-Aldrich; #F1804). LPL concentration was determined via Western blot using a standard curve of purified LPL protein (a kind gift from Michael Ploug). Western blots were performed using an anti-LPL monoclonal antibody (5D2) at a 1:400 dilution (Santa Cruz Biotechnology).

### Western blot

Protein samples were size fractionated on 12% SDS-PAGE gels and then transferred to a nitrocellulose membrane. Membranes were blocked with casein buffer (1% casein; Fisher Science Education). Primary antibodies were diluted in casein buffer + 0.1% Tween. Primary antibody dilutions were 1:3,000 for a mouse monoclonal antibody against EL (LIPG antibody clone 3C7; Lifespan Biosciences), 1:3,000 for a rabbit polyclonal antibody against Strep-tag II (Abcam), 1:5,000 for a mouse monoclonal antibody against V5 tag (R960-25; Invitrogen), 1:2,000 for a goat antibody against beta-actin (Abcam), and 1:400 for a mouse monoclonal antibody against LPL (5D2; Santa Cruz Biotechnology). After washing with PBS-T, membranes were incubated with Dylight680- or Dylight800-labeled secondary antibodies (Thermo Scientific) diluted 1:5,000 in casein buffer. After washing with PBS-T, antibody binding was detected using an Odyssey Clx Infrared Scanner (Li-Cor).

### EL activity assays

Phospholipase activity was monitored using the EnzChek Phospholipase A1 assay kit (Thermo Fisher Scientific) as described previously (26). EL (final concentration of ∼10 μg/ml) and ANGPTL proteins (final concentrations: 0–4,000 ng/ml ANGPTL3; 0–210 ng/ml ANGPTL8) were combined and incubated at 37°C (or at 4°C or 22°C for temperature studies) for 30 min (unless otherwise indicated). Following incubation, 50 μl of sample was mixed with 50 μl of substrate solution and were incubated at room temperature (approximately 20–22°C) for 30 min, reading fluorescence (485 nm excitation/515 nm emission) every 1 or 2 min with an Infinite F200 plate reader (Tecan). Relative phospholipase activity was determined by calculating the slope of the linear part of the curve (typically in the range between 5 and 25 min) and then subtracting out the slope of the blank (sample with no EL conditioned media).

To detect phospholipase activity on the cell surface, RHMVECs expressing EL were grown to confluency in a black 96-well clear bottom plate coated with fibronectin. After washing twice with 1× sterile PBS, cells were treated with 50 μl of ANGPTL3, ANGPTL3 + ANGPTL8, or control conditioned media for 30 min at 37°C. After incubation, 50 μl of substrate from the EnzChek Phospholipase A1 assay kit was added, and fluorescence (485 nm excitation/515 nm emission) was read at room temperature (approximately 20–22°C) every 1 or 2 min for 30 min with an Infinite F200 plate reader. Relative phospholipase activity was calculated by calculating the slope of the linear part of the curve (typically in the range between 5 and 25 min) and then subtracting out the slope of samples containing untransduced RHMVEC incubated with 50 μl control conditioned media.

### EL activity assay with HDL substrate

EL (final concentration of ∼60 μg/ml) and ANGPTL proteins (0–2,600 ng/ml ANGPTL3; 0–78 ng/ml ANGPTL8) were incubated together at 37°C for 30 min. Human HDL (Lee Biosolutions) was added to the sample to a final concentration of 62 mg/dl (cholesterol) and incubated at 37°C for an additional 30 min. After incubation, 50 μl of sample was loaded into a Greiner clear 96-well plate. NEFA levels in each sample were measured using a commercial kit (HR series NEFA-HR; Wako) and following the manufacturer's instructions. Briefly, 112.5 μl of NEFA reagent A (0.53 U/ml acyl-coenzyme A synthetase, 0.31 mmol coenzyme A, 4.3 mmol/l adenosine triphosphate, 1.5 mmol/l 4-aminoanitipyrine, 2.6 U/ml ascorbate oxidase, and 0.062% sodium azide) was added to each sample and incubated for 10 min at 37°C. 37.5 μl of NEFA reagent B (12 U/ml acyl-coenzyme A oxidase, 14 U/ml peroxidase) was then added, and samples were incubated for 5 min at 37°C. Absorbance was read at 560 and 670 nm with a Spectramax i3 (Molecular Devices). For data analysis, the values read at 670 nm were subtracted from 560 nm values. To calculate relative levels of NEFAs released, absorbance values from samples containing only control media and HDL were subtracted from all samples. The resulting values from samples containing EL, but no ANGPTL proteins, were then averaged, and this average value was set as 100%.

### LPL activity assay

Lipase activity was assayed using EnzCheck lipase fluorescent substrate (Molecular Probes) as described previously (27). Briefly, LPL (final concentration of ∼2.5 μg/ml) and ANGPTL proteins (final concentrations of 0–1,100 ng/ml ANGPTL3; 0–33 ng/ml ANGPTL8) were combined and incubated at 37°C for 30 min. 50 μl of the sample was then mixed with 25 μl 4× assay buffer (0.6 M NaCl, 80 mM Tris-HCl, pH 8.0, and 6% fatty acid-free BSA). 25 μl of substrate solution containing 2.48 μM EnzCheck lipase fluorescent substrate, 0.05% 3-(*N*,*N*-dimethylmyristylammonio) propanesulfonate zwittergent detergent (Acros Organics) in 1% methanol was then added to each sample. Samples were then incubated at 37°C with fluorescence (485 nm excitation/528 nm emission) read every 1 or 2 min for 30 min with a Spectramax i3 (Molecular Devices) or an Infinite F200 plate reader (Tecan). Relative LPL activity was determined by calculating the slope of the linear part of the curve (typically in the range between 5 and 25 min) and then subtracting out the slope of the blank (sample with no LPL conditioned media).

### EL binding assays

Binding between EL and various ANGPTL3 protein constructs was measured using the NanoBiT Split-Luciferase System (Promega) (28). Approximately 35 μg/ml of EL tagged with LargeBiT and 0.8 μg/ml of ANGPTL3 constructs tagged with smallBiT were incubated at 37°C for 30 min. 25 μl of the sample, 25 μl of serum-free DMEM, and 13 μl of NanoLuc Live Cell substrate (Promega) were added to a white opaque 96-well plate. The plate was incubated at 37°C, and luminescence was recorded after 12 min using a Spectramax i3 (Molecular Devices) or an Infinite F200 plate reader (Tecan). The signal of the blank (50 μl serum-free DMEM + 13 μl NanoLuc substrate) was subtracted from each reading, and relative luminescence units were plotted in a bar graph.

### LPL binding assays

Binding between LPL on the surface of endothelial cells and ANGPTL3 and ANGPTL3-ANGPTL8 complexes was measured using the NanoBiT Split-Luciferase System. RHMVECs stably expressing GPIHBP1 (described previously) were grown to confluency in an opaque-walled clear-bottom 96-well plate and washed with PBS. 50 μl of LargeBiT-tagged LPL (∼30 μg/ml) was added and allowed to bind cells at 4°C for 3 h. After this incubation, the cell layer was washed with PBS, and 50 μl of small-BiT–tagged ANGPTL3 with or without ANGPTL8 were added. After addition of 13 μl of NanoLuc Live Cell substrate (Promega), the plate was incubated at 37°C, and luminescence was recorded after 12 min using an Infinite F200 plate reader (Tecan). Relative luminescence units were normalized as a percentage of the smANGPTL3 (smA3) sample and plotted in a bar graph.

## Results

### Inhibition of EL by ANGPTL3

**Fig. 1:**
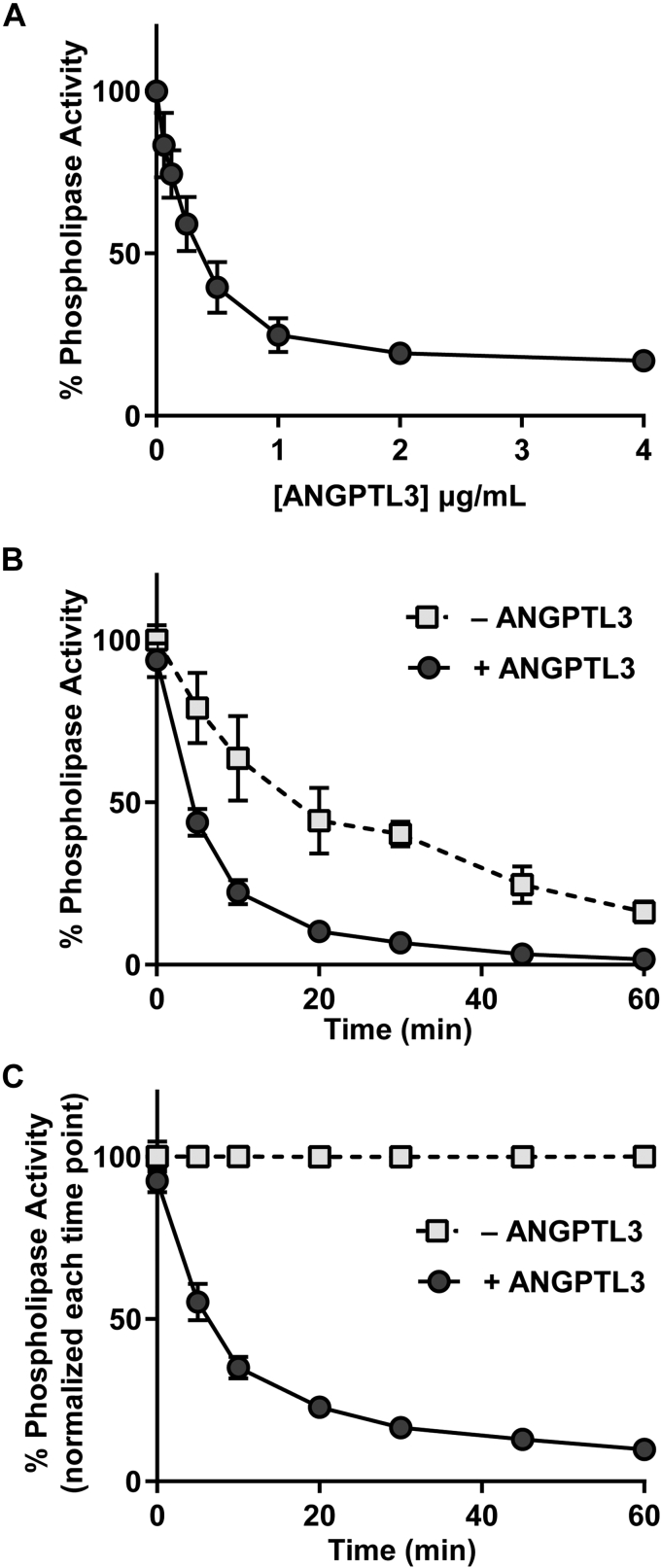
Inhibition of endothelial lipase by ANGPTL3. A: Phospholipase activity of EL after incubation with increasing concentrations of ANGPTL3 for 30 min at 37°C. Activity was normalized to control-treated EL. Points represent mean (±SD) of three independent experiments; each performed with biological duplicates. B and C: Phospholipase activity over time of EL incubated with or without 2 μg/ml ANGPTL3. Activity was normalized either to the activity of EL without ANGPTL3 (control treated) at time point 0 (B) or to the respective control-treated EL for each time point (C). Points represent mean (±SD) of three independent experiments; each performed with biological duplicates. ANGPTL3, angiopoietin-like 3; EL, endothelial lipase.

The ability of ANGPTL3 to inhibit EL has been reported previously (18). Consistent with these reports, when we incubated increasing concentrations of Strep-tagged ANGPTL3 with ∼10 μg/ml EL at 37°C for 30 min, we found that ANGPTL3 was able to inhibit the phospholipase activity of EL in a dose-dependent manner (Fig. 1A). Interestingly, in this assay, substochiometric levels of ANGPTL3 could maximally inhibit EL, suggesting that ANGPTL3-mediated inhibition might be catalytic.

**Fig. 2:**
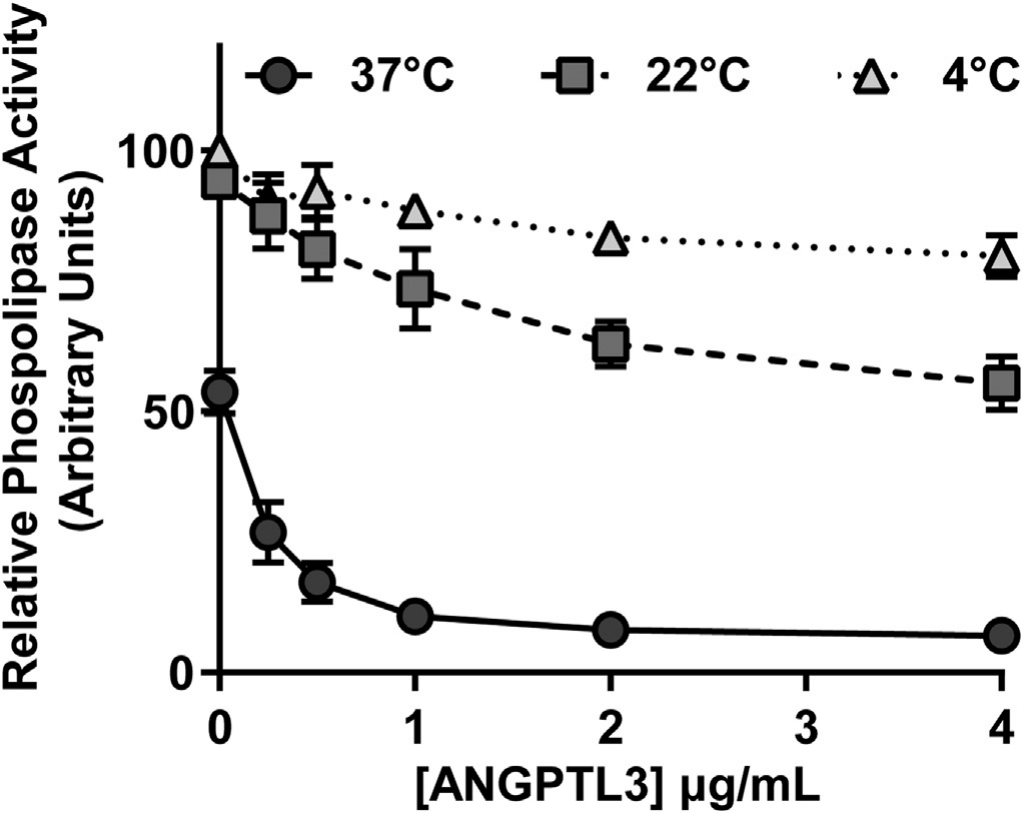
Temperature dependence of ANGPTL3-mediated EL inhibition. Relative phospholipase activity of EL (measured at room temperature) after incubation with increasing concentrations of ANGPTL3 for 30 min at 4, 22, or 37°C. Each data point represents mean (±SD) of three independent experiments; each performed with biological duplicates. ANGPTL3, angiopoietin-like 3; EL, endothelial lipase.

We next asked if inhibition of EL by ANGPTL3 was time dependent, a finding that might also support a catalytic mechanism of inhibition. To do so, we measured the phospholipase activity of EL at 0, 5, 10, 20, and 30 min after addition of 2 μg/ml ANGPTL3. Interestingly, in the absence of ANGPTL3, EL loses significant activity over time (Fig. 1B; open squares), a finding that is consistent with the spontaneous loss of activity observed with LPL (29, 30, 31). In the presence of ANGPTL3, EL activity is further reduced (Fig. 1B). When EL activity was normalized to untreated EL at each respective time point to correct for spontaneous loss of EL activity, ANGPTL3 treatment continued to reduce EL activity over time (Fig. 1C), again suggesting a catalytic mechanism of EL inhibition by ANGPTL3. Inhibition of EL by ANGPTL3 was also temperature dependent. When EL was incubated with ANGPTL3 at 4°C or 22°C for 30 min and then assayed for phospholipase activity, markedly less inhibition of phospholipase was observed compared with when EL and ANGPTL3 were incubated for 30 min at 37°C before assaying activity (Fig. 2). We also observed that spontaneous inactivation of EL was also much greater at 37°C than 4°C or 22°C as judged by relative phospholipase activity in the absence of ANGPTL3 (Fig. 2; 0 μg/ml concentration).

**Fig. 3:**
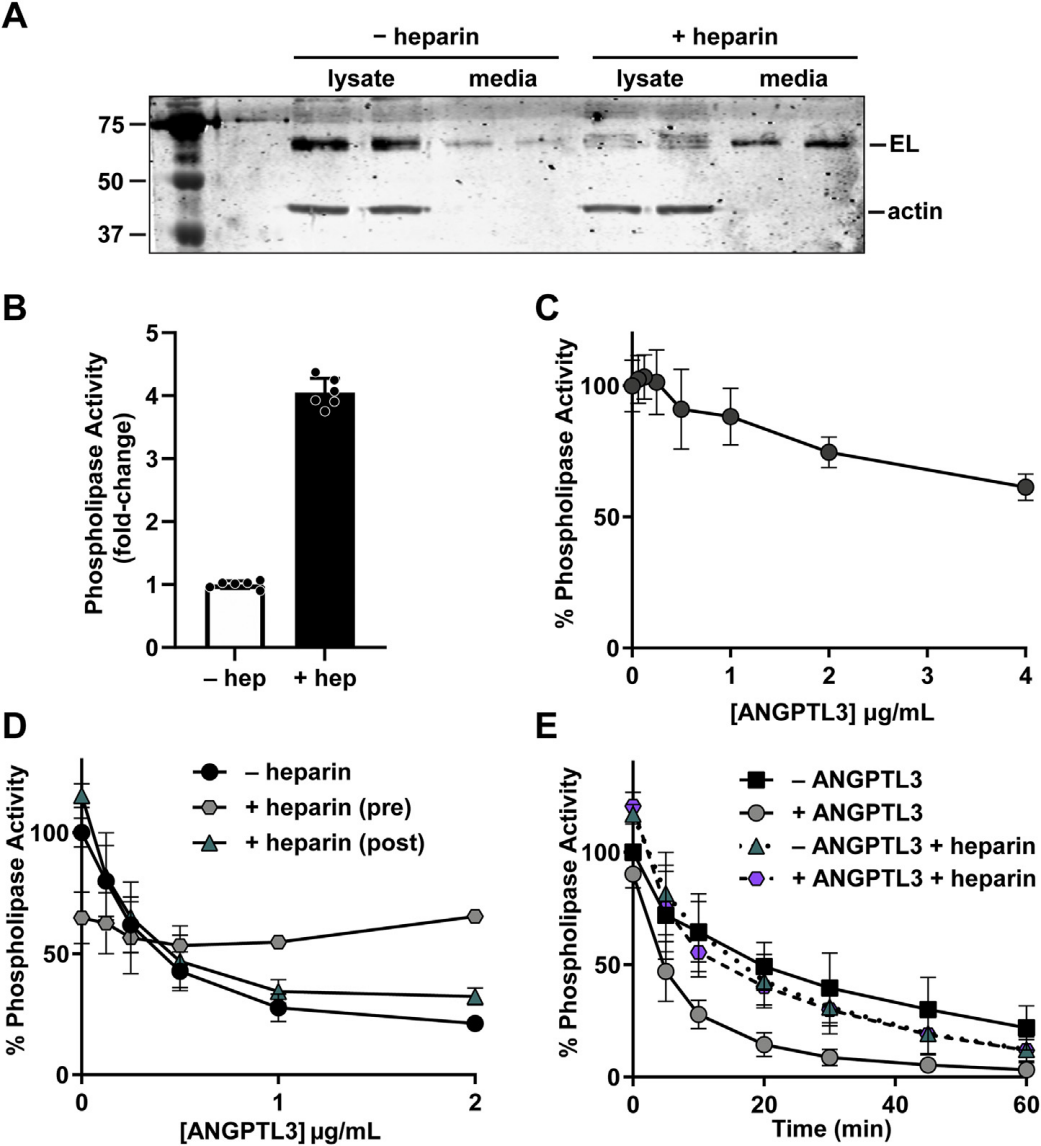
ANGPTL3 inhibition of EL bound to cells or in the presence of heparin. A: Representative Western blot of media and lysate collected from rat heart microvessel endothelial cells (RHMVECs) expressing EL and treated with or without 0.1 U/ml heparin for 24 h. B: Phospholipase activity of media collected from EL-expressing RHMVECs treated with or without 0.1 U/ml heparin for 24 h. Points represent mean (±SD) of two independent experiments, each with three independent activity measurements. C: Phospholipase activity of EL-expressing RHMVECs after incubation with increasing concentrations of ANGPTL3 at 37°C for 30 min. Each data point represents mean (±SD) of three independent experiments performed with biological triplicates. Activity was normalized to untreated EL-expressing RHMVECs. D: Phospholipase activity of EL after incubation with (+ heparin (pre)) or without (− heparin) 1 U/ml heparin and with increasing concentrations of ANGPTL3 for 30 min at 37°C. Following incubation, a portion of the samples without heparin were then treated with 1 U/ml heparin (+ heparin (post)) and also assayed for phospholipase activity. Points represent mean (±SD) of three independent experiments performed with biological triplicates. E: Phospholipase activity over time of EL incubated with or without 1 U/ml heparin and with or without 2 μg/ml ANGPTL3 at 37°C. Points represent mean (±SD) of three independent experiments performed with biological triplicates. ANGPTL3, angiopoietin-like 3; EL, endothelial lipase.

In vivo, EL is bound to the surface of endothelial cells via heparan sulfate proteoglycans (HSPGs) (32). We found that EL likewise remained bound to the surface of endothelial cells in cell culture (Fig. 3A). Both EL protein and phospholipase activity could be released into the media by treatment with heparin (Fig. 3A, B), suggesting that EL was bound to surface HSPGs. LPL, a lipase in the same family as EL, is protected from inhibition by ANGPTL proteins when bound to endothelial cells by its transporter, GPIHBP1 (21, 24, 33, 34, 35). We asked if HSPG-bound EL on the surface of endothelial cells were protected from ANGPTL3 inhibition in a similar way. When EL is in solution, we found that ANGPTL3 inhibited EL with an IC_50_ of approximately 260 ng/ml (Fig. 1A). However, when we incubated ANGPTL3 with EL bound to endothelial cells, we found that the ability of ANGPTL3 to inhibit was dramatically reduced (Fig. 3C), strongly suggesting that cell-bound EL is much less susceptible to ANGPTL3 inhibition.

### Heparin protects EL from ANGPTL3 inhibition but cannot reverse inhibition

Given the difference in the ability of ANGPTL3 to inhibit cell-bound EL versus unbound EL, we reasoned that the interaction of EL with the heparan-sulfate moieties on HSPGs might provide protection to EL. It has been previously reported that heparin protects LPL from ANGPTL3 inhibition (33). Thus, we asked if we could replicate the protection of cell-bound EL with heparin. EL was incubated with increasing concentrations of ANGPTL3 alone or in the presence of 1 U/ml heparin. Interestingly, in the absence of ANGPTL3, incubation of EL with heparin reduced phospholipase activity (Fig. 3D). However, in the presence of 1 U/ml heparin, ANGPTL3 had no effect on phospholipase activity (Fig. 3D; gray hexagons), suggesting that heparin protects EL from ANGPTL3. Because the EL used in our assays was collected in heparin, a small amount of heparin (0.008 U/ml) was present in all assays. To ensure that this residual heparin did not interfere with ANGPTL3 inhibition, we compared the ability of ANGPTL3 to inhibit EL collected normally (in the presence of heparin) with inhibition of EL collected without heparin (collection is inefficient but possible). We observed no difference in EL inhibition (supplemental Fig. S2A). Adding back heparin to EL that had been collected in the absence of heparin such that the final assay concentration of heparin was 0.008 U/ml also had no effect on ANGPTL3 inhibition (supplemental Fig. S2B).

To determine if heparin might also reverse ANGPTL3 inhibition, samples that had been incubated with increasing concentrations of ANGPTL3 for 30 min were then treated with 1 U/ml heparin and retested for phospholipase activity. As expected, before heparin treatment, ANGPTL3 inhibited EL in a dose-dependent manner (Fig. 3D; black circles). Treating with heparin after ANGPTL3 inhibition resulted in no change in dose-dependent inhibition (Fig. 3D; teal triangles), indicating that heparin cannot reverse ANGPTL3 inhibition.

To further investigate the effects of heparin on EL activity, we conducted a phospholipase activity time course in the presence or the absence of 1 U/ml heparin. EL conditioned media were incubated with or without 2 μg/ml ANGPTL3 at 37°C in the presence or absence of 1 U/ml heparin for 0–60 min. As before, in the absence of ANGPTL3 or heparin, EL lost activity over time (Fig. 3E; black squares). As expected, treatment with ANGPTL3 greatly decreased EL activity, consistent with ANGPTL3-mediated inhibition (Fig. 3E; gray circles). In the presence of heparin, phospholipase activity decreased at a faster rate than in the absence of heparin with a half-life of ∼15 min compared with ∼25 min for untreated EL (Fig. 3E; teal triangles). However, in the presence of heparin, the addition of ANGPTL3 had no added effect on phospholipase activity (Fig. 3E; purple hexagons). Together, these data suggest that 1 U/ml heparin has a detrimental effect on EL activity, but at the same time, it protects EL from ANGPTL3 inhibition.

### The LPL-interaction domain of ANGPTL3 is necessary for the inhibition of EL

**Fig. 4:**
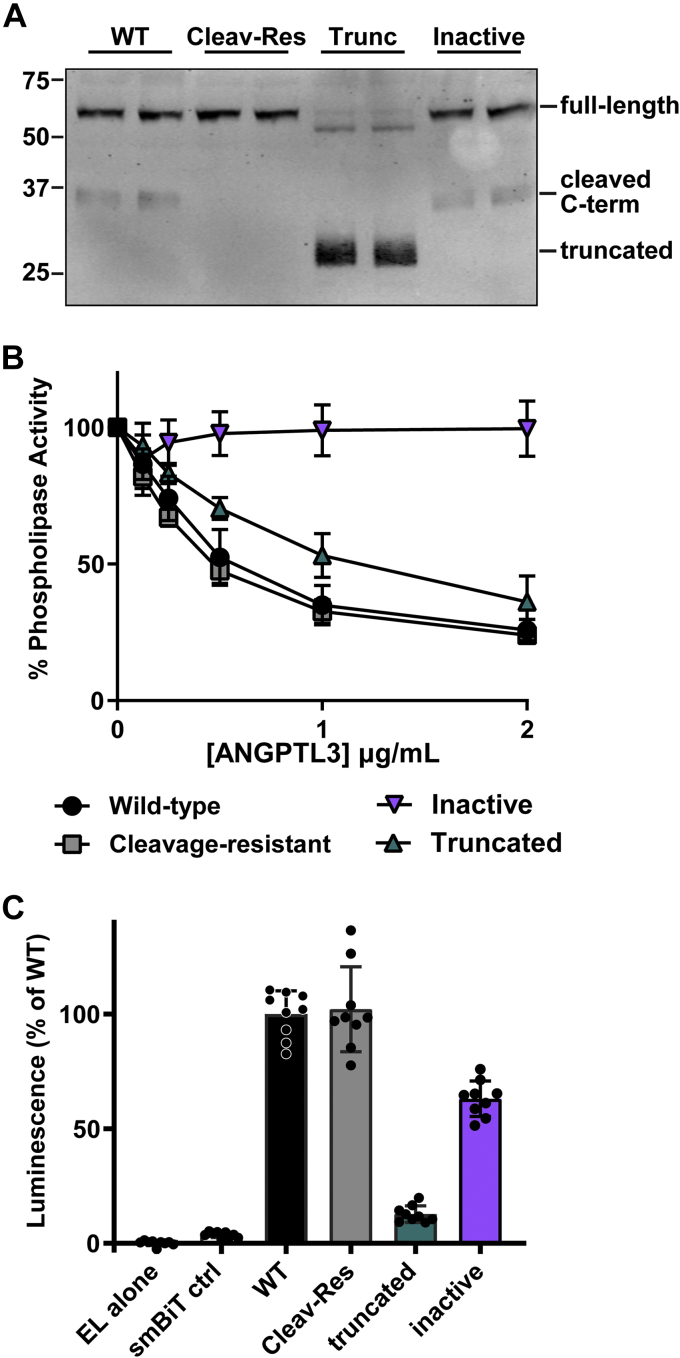
Inhibition of EL by inactive, truncated, and cleavage-resistant ANGPTL3. A: Western blot showing levels of wild-type (WT), cleavage-resistant (Cleav-Res), truncated (trunc), and inactive ANGPTL3 protein in conditioned media. B: Phospholipase activity of EL after incubation with increasing concentrations of wild-type, truncated, cleavage-resistant, or inactive ANGPTL3 at 37°C for 30 min. Points represents mean (±SD) of three independent experiments performed with biological duplicates. C: Relative protein binding of wild-type, cleavage-resistant, truncated, and inactive ANGPTL3 as indicated by reconstituted luciferase activity. LargeBiT-EL was incubated with 0.8 μg/ml of smallBiT-tagged wild-type, truncated, cleavage-resistant, or inactive ANGPTL3 at 37°C for 30 min. Luminescence was measured after adding luciferase substrate. Bar graph represents mean (±SD) of three independent experiments performed with biological triplicates. ANGPTL3, angiopoietin-like 3; EL, endothelial lipase.

The SE1/LPL inhibitory domain of ANGPTL3 (amino acids 32–55) is important for the binding and inhibition of LPL (36, 37). To ask if this region is also necessary for the inhibition of EL by ANGPTL3, we generated three mutations in the LPL inhibitory domain—N48A, Q53A, and H55A. These mutations replace the three polar residues thought to be essential for LPL binding and inhibition (36). Although this mutant ANGPTL3, which we refer to as “inactive,” was expressed and secreted at normal levels (Fig. 4A), it had no ability to inhibit EL (Fig. 4B).

### Inhibition of EL by truncated and cleavage-resistant ANGPTL3

ANGPTL3 contains a cleavage site in the linker region between the N-terminal coiled-coil domain and the C-terminal fibrinogen-like domain (38). The N-terminal coiled-coil region of ANGPTL3 has been shown to be necessary and sufficient for EL inhibition (18, 39). It has been suggested that the cleaved N-terminal domain might inhibit EL more efficiently (39). To explore this issue, we generated a truncated ANGPTL3 containing only the N-terminal region (amino acids 1–221) and a cleavage-resistant ANGPTL3 (RAPR 221–224 GSGS) and compared their ability to inhibit EL to that of wild-type EL. As expected, the truncated N-terminal construct produced primarily truncated ANGPTL3 protein, cleavage-resistant ANGPTL3 only produced full-length protein, and wild-type ANGPTL3 produced both full-length and cleaved protein (Fig. 4A). We tested the ability of these proteins to inhibit EL by incubating EL conditioned media with increasing concentrations of wild-type, truncated, and cleavage-resistant ANGPTL3 at 37°C for 30 min. We found that cleavage-resistant ANGPTL3 and wild-type ANGPTL3 inhibited EL equally well (Fig. 4B). Interestingly, although truncated ANGPTL3 retained some ability to inhibit EL, inhibition was reduced compared with wild-type ANGPTL3 (Fig. 4B). These results suggest that the C-terminal region of ANGPTL3 may be necessary for maximal inhibition of EL.

### The binding of mutant ANGPTL3s to EL

We next asked if the relative abilities of cleavage-resistant, truncated, and inactive ANGPTL3 to inhibit EL activity were a reflection of differences in binding. To do so, we used the NanoBiT Split-Luciferase System (28). In this assay, protein pairs are tagged with either LargeBiT or smallBiT tags. When protein pairs come together, the combination of the LargeBiT tag and the smallBiT tag reconstitute luciferase activity in proportion to the level of binding (28, 40). We tagged EL with LargeBiT and each ANGPTL3 construct with the smallBiT. Not surprisingly, we found that cleavage-resistant ANGPTL3 bound to EL at nearly identical levels to wild-type ANGPTL3 (Fig. 4C). Interestingly, our inactive ANGPTL3 protein retained a significant ability to bind EL even though it has no ability to inhibit EL (Fig. 4C). Conversely, the ability of truncated ANGPTL3 to bind EL was significantly reduced, but this protein still retained the ability to inhibit EL (Fig. 4C). These results suggest that ANGPTL3 binding and ANGPTL3 inhibition may be separable activities.

### The role of ANGPTL8 in the inhibition of EL

**Fig. 5:**
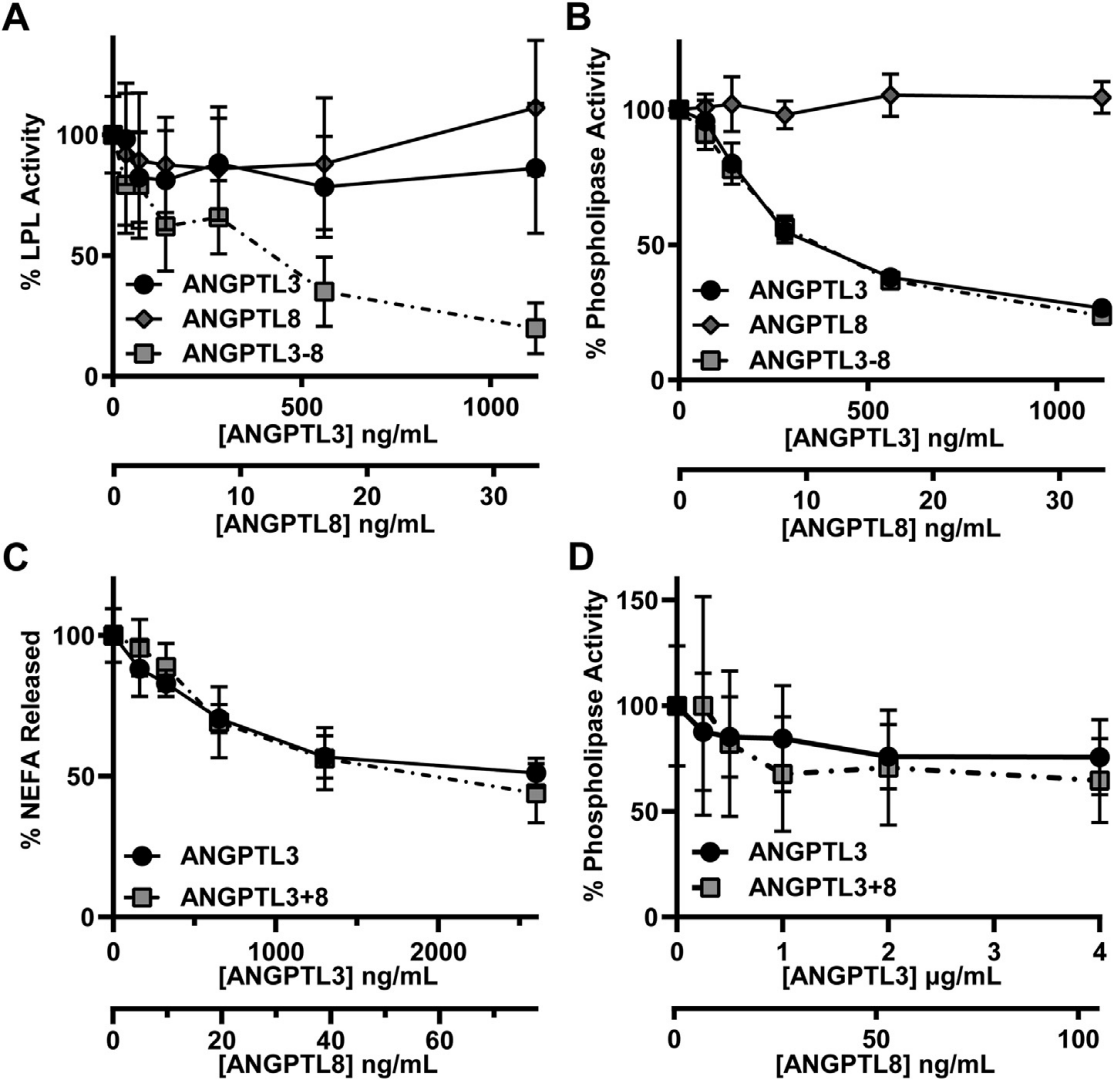
Contribution of ANGPTL8 to EL inhibition. A: Lipase activity of LPL after incubation with increasing concentrations of ANGPTL3, ANGPTL8, or ANGPTL3-ANGPTL8 complexes for 30 min at 37°C. Points represent mean (±SD) of five independent experiments performed with biological duplicates. B: Phospholipase activity of EL after incubation with increasing concentrations of ANGPTL3, ANGPTL8, or ANGPTL3-ANGPTL8 complexes for 30 min at 37°C. Points represent mean (±SD) of three independent experiments; each performed with biological duplicates. C: Phospholipase activity of EL incubated with increasing concentrations of ANGPTL3 or ANGPTL3-ANGPTL8 complexes for 30 min at 37°C as measured by the release of NEFAs from HDL. Points represent mean (±SD) of three independent experiments performed with biological duplicates. NEFA release was normalized to the 0 μg/ml ANGPTL sample, which was set to 100%. D: Phospholipase activity of EL-expressing RHMVECs after incubation with the indicated concentrations of ANGPTL3 or ANGPTL3-ANGPTL8 complexes at 37°C for 30 min. Data points represent mean (±SD) of three independent experiments performed with biological triplicates. Activity was normalized to untreated EL-expressing RHMVECs. ANGPTL3, angiopoietin-like 3; ANGPTL8, angiopoietin-like 8; EL, endothelial lipase; RHMVECs, rat heart microvessel endothelial cells.

ANGPTL8 plays a vital role in the inhibition of LPL by ANGPTL3 (15, 21, 22, 23). We have previously shown that ANGPTL8-ANGPTL3 complexes bind and inhibit LPL activity significantly better than either ANGPTL3 or ANGPTL8 alone (21). We asked whether ANGPTL8 also modulates the ability of ANGPTL3 to inhibit EL. Consistent with our previous finding, when we treated LPL with ANGPTL3, ANGPTL8, or ANGPTL3 and ANGPTL8 that had been coexpressed, we found that ANGPTL3-ANGPTL8 complexes inhibited LPL far better than ANGPTL3 or ANGPTL8 alone (Fig. 5A). However, when we treated EL with ANGPTL3 alone, ANGPTL8 alone, or coexpressed ANGPTL3-ANGPTL8, the results were quite different. We found that ANGPTL8 alone had no inhibitory effect on EL, whereas, as we have shown previously in this study, ANGPTL3 alone was able to efficiently inhibit EL (Fig. 5B). Importantly, ANGPTL3-ANGPTL8 complexes showed no increase in their ability to inhibit EL compared with ANGPTL3 alone, suggesting that the presence of ANGPTL8 had no effect on EL inhibition (Fig. 5B). Similar results were obtained when EL activity was assessed by measuring the release of nonesterified fatty acids from human HDL (Fig. 5C). We then asked if perhaps ANGPTL8 improved the ability of ANGPTL3 to inhibit EL bound to endothelial cells. However, we found that both ANGPTL3 alone and ANGPTL3-ANGPTL8 complexes inhibited cell-bound EL to a similar degree even at supraphysiological concentrations (Fig. 5D).

### The effect of an R59W mutation in ANGPTL8 on lipase inhibition

**Fig. 6:**
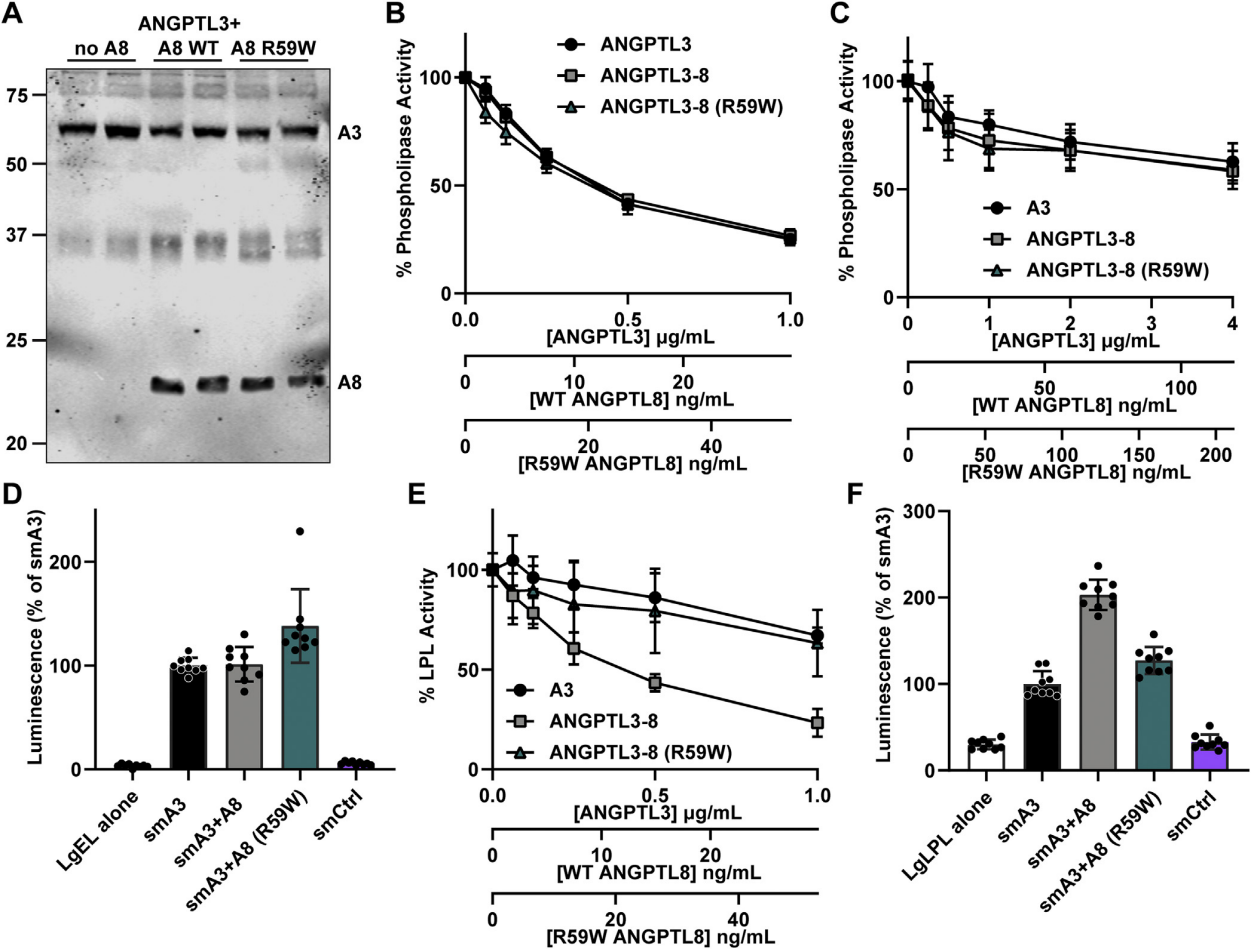
Effect of ANGPTL8 R59W on EL and LPL inhibition. A: Western blot showing levels of ANGPTL3 and ANGPTL8 in conditioned media after transfection with ANGPTL3 alone (no A8) or cotransfected with wild-type ANGPTL8 (A8 WT) or ANGPTL8 R59W (A8 R59W). B: Phospholipase activity of EL after incubation for 30 min at 37°C with increasing concentrations of ANGPTL3, ANGPTL3-ANGPTL8 complexes, or ANGPTL3-ANGPTL8 (R59W) complexes. Points represent mean (±SD) of three independent experiments; each performed with biological duplicates. C: Phospholipase activity of EL-expressing RHMVECs after incubation with the indicated concentrations of ANGPTL3, ANGPTL3-ANGPTL8 complexes, or ANGPTL3-ANGPTL8 (R59W) complexes at 37°C for 30 min. Data points represent mean (±SD) of three independent experiments performed with biological triplicates. Activity was normalized to untreated EL-expressing RHMVECs. D: Relative protein binding of ANGPTL3, ANGPTL3-ANGPTL8 complexes, and ANGPTL3-ANGPTL8 (R59W) complexes as indicated by reconstituted luciferase activity. LargeBiT-EL was incubated with 0.6 μg/ml of smallBiT-tagged ANGPTL3, ANGPTL3-ANGPTL8 complexes ([A8] = 64 ng/ml), or ANGPTL3-ANGPTL8 (R59W) complexes ([A8] = 48.8 ng/ml) at 37°C for 30 min. Luminescence was measured after adding luciferase substrate. Bar graph represents mean (±SD) of three independent experiments performed with biological triplicates. E: Lipase activity of LPL after incubation for 30 min at 37°C with increasing concentrations of ANGPTL3, ANGPTL3-ANGPTL8 complexes, or ANGPTL3-ANGPTL8 (R59W) complexes. Points represent mean (±SD) of three independent experiments performed with biological duplicates. F: Relative protein binding of ANGPTL3, ANGPTL3-ANGPTL8 complexes, and ANGPTL3-ANGPTL8 (R59W) complexes as indicated by reconstituted luciferase activity. LargeBiT-LPL was bound to GPIHBP1-expressing RHMVECs. After washing off unbound LPL, 0.6 μg/ml of smallBiT-tagged ANGPTL3, ANGPTL3-ANGPTL8 complexes ([A8] = 64 ng/ml), or ANGPTL3-ANGPTL8 (R59W) complexes ([A8] = 48.8 ng/ml) were added, and luminescence was measured after adding luciferase substrate. Bar graph represents mean (±SD) of three independent experiments performed with biological triplicates. ANGPTL3, angiopoietin-like 3; ANGPTL8, angiopoietin-like 8; EL, endothelial lipase; RHMVECs, rat heart microvessel endothelial cells.

In humans, a mutation that codes for tryptophan at amino acid 59 of ANGPTL8 (R59W) is associated with lower HDL-C levels than that found in individuals with the normal arginine at that position (41, 42). The cause of lower HDL-C levels in these individuals is not known, but one possibility is that ANGPTL8 R59W decreases the ability of ANGPTL3 to inhibit EL, thus increasing EL activity and reducing HDL-C levels. Such a finding would be intriguing given our data suggesting that ANGPTL8 does not normally modulate the ability of ANGPTL3 to inhibit EL. To test this idea, we assessed the ability of ANGPTL3, ANGPTL3-ANGPTL8 complexes, and ANGPTL3-ANGPTL8 (R59W) complexes to inhibit EL in vitro. When coexpressed with ANGPTL3, ANGPTL8 R59W was secreted normally from cells (Fig. 6A). As ANGPTL8 requires ANGPTL3 for efficient secretion (21), these data suggest that ANGPTL8 R59W retains the ability to bind ANGPTL3. We found no difference in the ability of ANGPTL3-ANGPTL8 (R59W) complexes to inhibit EL compared with ANGPTL3 or wild-type ANGPTL3-ANGPTL8 complexes (Fig. 6B). We also tested if ANGPTL8 (R59W) would affect the ability of the ANGPTL3 to inhibit EL when bound to the surface of endothelial cells. As before, EL bound to endothelial cells was largely resistant to ANGPTL3 inhibition, but again, neither ANGPTL8 nor ANGPTL8 (R59W) altered inhibition (Fig. 6C). Next, we assessed the ability of ANGPTL3 alone and ANGPTL3-ANGPTL8 complexes to bind EL using the NanoBiT Split-Luciferase System. As expected, ANGPTL3 bound robustly to EL and wild-type ANGPTL8 did not alter this binding (Fig. 6D). ANGPTL8 (R59W) appeared to slightly increase the binding of ANGPTL3 to EL (Fig. 6D). The biological significance of this increased binding is not clear as it does not appear to alter EL inhibition. Finally, we assessed if ANGPTL3-ANGPTL8 complexes containing ANGPTL8 (R59W) had altered ability to inhibit LPL. Unlike the situation with EL, we saw a significant reduction in the ability of ANGPTL3-ANGPTL8 complexes to inhibit LPL when ANGPTL8 harbored the R59W mutation (Fig. 6E). This decrease in inhibition could be attributed to a change in binding, as binding to LPL was reduced to the level of ANGPTL3 alone when ANGPTL3-ANGPTL8 complexes contained ANGPTL8 with the R59W mutation (Fig. 6F).

## Discussion

In this study, we investigated the ability of ANGPTL3 to inhibit EL and assessed the role of ANGPTL8 in the regulation of EL by ANGPTL3. We found that ANGPTL3 inhibition of EL is dose dependent, temperature dependent, and time dependent. Interestingly, when EL is bound to the surface of the cell or when heparin is present, ANGPTL3-mediated inhibition is blunted. We also found that neither ANGPTL8 nor a common ANGPTL8 variant (R59W) affected the ability of ANGPTL3 to inhibit EL. The R59W ANGPTL8 variant, when in complex with ANGPTL3, did have reduced ability to inhibit LPL.

The binding of EL to HSPGs on the surface of cells has been previously reported (32). We likewise found that EL binds to the surface of endothelial cells, likely through its interaction with HSPGs. Our data indicate that HSPGs and heparin protect EL from ANGPTL3 inhibition. This finding has important physiological implications. The protection of EL by HSPGs suggests that EL bound to the surface of the vasculature is protected from ANGPTL3, whereas circulating EL is not. Such a situation could potentially ensure that only EL that is cell bound and can specifically target delivery of HDL-derived fatty acids to a specific tissue is enzymatically active.

Previously, we and others have reported that ANGPTL4, a related ANGPTL family member that regulates LPL, has increased ability to inhibit LPL when truncated to only the N-terminal domain (24, 43, 44). However, we also have reported that the truncated N-terminal domain of ANGPTL3, either alone or in complex with ANGPTL8, has reduced ability to inhibit LPL compared with full-length ANGPTL3 (21). Here, we likewise report that full-length ANGPTL3 more efficiently inhibits EL than the N-terminal domain by itself, suggesting that the C-terminal fibrinogen-like domain does contribute in some manner to both LPL and EL inhibition.

Several mechanisms have been proposed for the inhibition of LPL by ANGPTL3, including suppression of catalytic activity (45), unfolding the catalytic triad of LPL (35), increasing the cleavage of LPL by secreted proteases (46), and dissociating the active dimers of LPL into inactive monomers (36). Which, if any, of these mechanisms are accurate remain unclear, nor has the mechanism by which ANGPTL3 inhibits EL been directly investigated. Although our data do not fully delineate the mechanism of EL inhibition, they do provide some important information. We observed that an inactive version of ANGPTL3 could still bind EL, but could not inhibit, suggesting a mechanism where binding alone is not sufficient for inhibition. We also found that substochiometric levels of ANGPTL3 could efficiently inhibit EL, that ANGPTL3-mediated inhibition increases over time, and that inhibition was temperature dependent. Finally, we observed that heparin can prevent ANGPTL3 inhibition of EL but cannot rescue inhibition after it has occurred. Together, these data support a catalytic inhibitory mechanism in which one ANGPTL3 molecule (or oligomer) is able to inactivate multiple molecules of EL and that once inactivated, EL remains inactive even when ANGPTL3 dissociates. Such a mechanism has been described for ANGPTL4 inhibition of LPL (35, 47). However, our data are not definitively conclusive. Time and temperature dependence might be explained by slow and temperature-dependent binding kinetics. A more detailed mechanistic study will be required to conclusively define the mechanism of action of ANGPTL3.

Several laboratories, including our own, have shown that ANGPTL3 inhibits LPL with much more potency when it is in complex with ANGPTL8 (15, 21, 22, 23). We investigated whether the same was true for the inhibition of EL. Our data clearly indicate that ANGPTL8 does not enhance the ability of ANGPTL3 to bind or inhibit EL. This finding suggests that ANGPTL8 serves as an LPL-specific adaptor. How ANGPTL3-ANGPTL8 inhibition of LPL differs structurally and mechanistically from ANGPTL3-mediated inhibition of EL is not clear. Haller *et al.* (15) found that the lipase inhibitory domain of ANGPTL8 was critical for inhibition of LPL and was capable of LPL inhibition even when the inhibitory domain of ANGPTL3 was mutated, suggesting that ANGPTL8 does not simply promote the binding of ANGPTL3 to LPL. Further structural and mechanistic studies will be required to explain the importance of ANGPTL8 in LPL inhibition but its dispensableness for EL inhibition. Although ANGPTL8 does not appear to directly alter the ability of ANGPTL3 to inhibit EL, it is possible that by allowing ANGPTL3 to bind more readily to LPL, ANGPTL8 shifts the amount of ANGPTL3 available for EL inhibition and thus indirectly modulates EL activity. Careful competition studies will be needed to show that this is the case.

Our results with ANGPTL8 R59W are somewhat paradoxical. In humans, the R59W mutation is associated with lower HDL-C levels in some ethnic groups but is not associated with TG levels (41, 42, 48, 49). These findings suggest that ANGPTL8 R59W might affect EL inhibition by ANGPTL3 but not LPL inhibition. We found the opposite. ANGPTL8 R59W did not alter the ability of ANGPTL3 to inhibit EL. However, LPL binding and inhibition was greatly reduced when ANGPTL8 R59W, rather than wild-type ANGPTL8, was in complex with ANGPTL3. Our data suggest that the effect of R59W on HDL-C levels is not a result of an altered ability of ANGPTL3 to inhibit EL. It is possible that in vivo, ANGPTL8 R59W alters the levels of functional ANGPTL3 in a way that could not be observed in our assays, or that its effect on HDL-C levels is indirect. Given our observation that ANGPTL8 R59W greatly reduced the ability of ANGPTL3-ANGPTL8 complexes to inhibit LPL, it is not clear why no TG phenotype has been observed for this mutation. Potentially, the physiological effect of ANGPTL8 R59W on TGs in vivo has been masked by the fact that studies of this SNP have all measured lipid levels from fasted individuals. ANGPTL8 is refeeding induced, and the effect of ANGPTL8 deficiency on plasma TG levels is much more prominent in the fed state (14, 42, 50, 51, 52). More studies, such as measuring patient's TG levels in the refed state or introducing this mutation into mouse models, may be necessary to fully elucidate the effect of this mutation on TG metabolism in vivo.

Overall, our results provide important details pertaining to the inhibition of EL by ANGPTL3. As clinical studies continue to test the efficacy of targeting ANGPTL3 to treat dyslipidemia, investigations into the mechanisms and physiological roles of ANGPTL3-mediated lipase inhibition continue to be important. ANGPTL3 clearly regulates HDL-C levels by inhibiting EL, but in the future, it will be imperative to understand how inhibition of EL by ANGPTL3 alters HDL function and its antiatherosclerotic properties. The newly uncovered role of ANGPTL3 and EL in modulating the conversion of VLDL remnants to LDL (10) also warrants further investigation.

## Data availability

All data for this study are contained within this article and in the supplemental data.

## Supplemental data

This article contains supplemental data.

## Conflict of interest

The authors declare that they have no conflicts of interest with the contents of this article.
