# Supplementary material for: Angiopoietin-like 3 inhibition of endothelial lipase is not modulated by angiopoietin-like 8
Source: J Lipid Res. 2021 Aug 27;62:100112. doi: 10.1016/j.jlr.2021.100112 (PMC8456055; doi:10.1016/j.jlr.2021.100112)
Supplement: Supplemental Figures S1 and S2 [file mmc1.pdf]

Supplemental Material

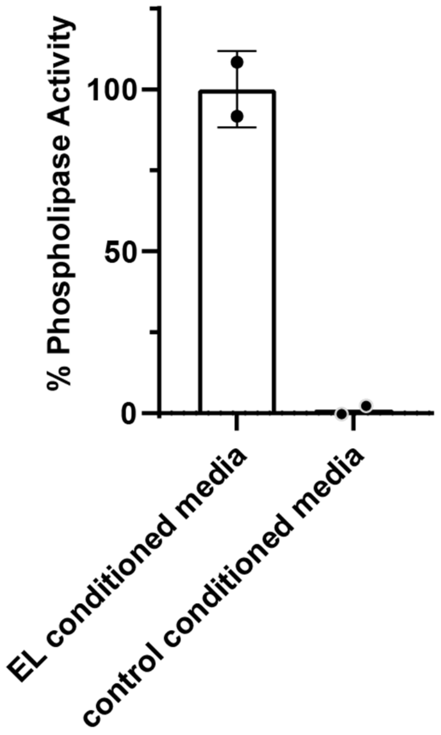

Supplemental Figure S1. Phospholipase activity in control conditioned media. Phospholipase activity of EL conditioned media and conditioned media from untransduced HEK 293T cells. Media and collection conditions were the same for all samples. Points represent mean (±SD) of two independent experiments each performed with technical duplicates.

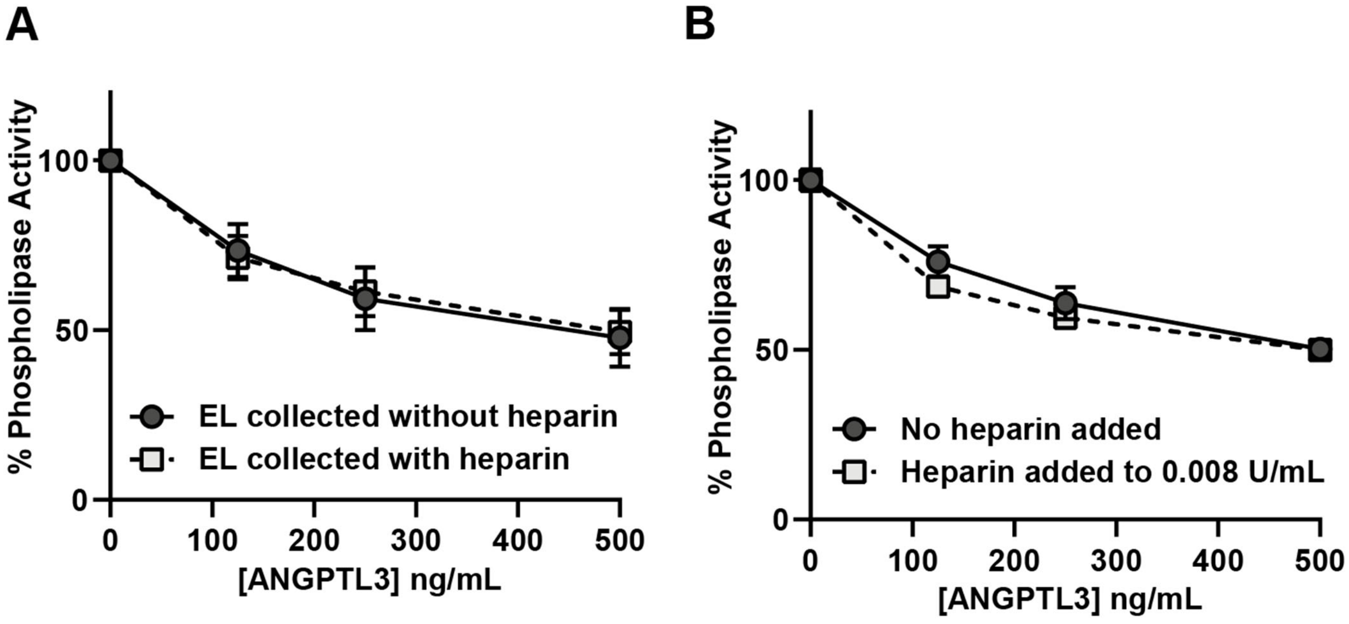

Supplemental Figure S2. Effect of low levels of heparin on ANGPTL3-mediated EL inhibition. (A) EL-containing conditioned media was collected from cells in the presence or absence of 0.1 U/ml heparin. EL from each sample was diluted such that all samples were matched for phospholipase activity. Samples were then incubated with the indicated concentrations of ANGPTL3 for 10 minutes at 37°C. Following incubation, phospholipase activity of EL was assayed. Points represent mean (±SD) of two independent experiments each performed with biological duplicates. **(B)** EL-containing conditioned media was collected from cells in the absence of heparin. To some of this conditioned media, heparin was added such that the final concentration, when incubated with ANGPTL3, would be 0.008 U/ml. Following incubation with the indicated concentrations of ANGPTL3 for 10 minutes at 37°C, phospholipase activity of EL was measured and plotted. Points represent mean (±SD) of two independent experiments each performed with biological duplicates.
